# Effectiveness of Probiotics, Prebiotics, and Symbiotic Supplementation in Cystic Fibrosis Patients: A Systematic Review and Meta-Analysis of Clinical Trials

**DOI:** 10.3390/medicina61030489

**Published:** 2025-03-12

**Authors:** Freiser Eceomo Cruz Mosquera, Claudia Lorena Perlaza, Anisbed Naranjo Rojas, Saray Murillo Rios, Alejandra Carrero Gallego, Sara Isabel Fischersworring, Juan Sebastián Rodríguez, Yamil Liscano

**Affiliations:** Grupo de Investigación en Salud Integral (GISI), Department of Health Sciences Faculty, Universidad Santiago de Cali, Cali 760035, Colombia; lorena.perlaza00@usc.edu.co (C.L.P.); anisbed.naranjo00@usc.edu.co (A.N.R.); sarayrios00@usc.edu.co (S.M.R.); diana.carrero00@usc.edu.co (A.C.G.); sarafischersworring@gmail.com (S.I.F.); juan.rodriguez24@usc.edu.co (J.S.R.); yamil.liscano00@usc.edu.co (Y.L.)

**Keywords:** cystic fibrosis, probiotics, dysbiosis, microbiota, systematic review

## Abstract

*Background and Objectives*: Cystic fibrosis (CF), caused by CFTR gene mutations, primarily affects the respiratory and gastrointestinal systems. Microbiota modulation through probiotics, prebiotics, or synbiotics may help restore microbial diversity and reduce inflammation. This study aimed to evaluate their efficacy in CF. *Materials and Methods:* A systematic review and meta-analysis of randomized controlled trials (RCTs) published between 2000 and 2024 was conducted in Cochrane, ScienceDirect, Web of Science, LILAC, BMC, PubMed, and SCOPUS following PRISMA guidelines. Methodological quality was assessed using the Jadad scale, and RevMan 5.4^®^ estimated effects on pulmonary function (FEV_1_), exacerbations, hospitalizations, quality of life, and inflammatory markers. *Results*: Thirteen RCTs (*n* = 552), mostly in pediatric populations, were included. Most examined probiotics (e.g., *Lactobacillus rhamnosus GG*, *L. reuteri*), while four used synbiotics. Several studies reported reduced fecal calprotectin and proinflammatory interleukins (e.g., IL-6, IL-8), suggesting an anti-inflammatory effect. However, no significant differences were observed regarding hospitalizations or quality of life. Additionally, none of the studies documented serious adverse events associated with the intervention. The meta-analysis showed no significant decrease in exacerbations (RR = 0.81; 95% CI = 0.48–1.37; *p* = 0.43) or improvements in FEV_1_ (MD = 4.7; 95% CI = −5.4 to 14.8; *p* = 0.37), even in subgroup analyses. Sensitivity analyses did not modify the effect of the intervention on pulmonary function or exacerbation frequency, supporting the robustness of the findings. *Conclusions*: Current evidence suggests that probiotics or synbiotics yield inconsistent clinical benefits in CF, although some reduction in inflammatory markers may occur. Larger, multicenter RCTs with longer follow-up are needed for clearer conclusions. Until more definitive evidence is available, these supplements should be considered experimental adjuncts rather than standard interventions for CF management.

## 1. Introduction

Cystic fibrosis (CF) is a monogenic disorder caused by mutations in the *Cystic Fibrosis Transmembrane Conductance Regulator (CFTR)* gene on chromosome 7, characterized by substantial clinical heterogeneity [1,2,3]. To date, over 2110 mutations of the *CFTR* gene have been identified, but only 401 have been confirmed to cause CF, as they are associated with a reduced or absent functional CFTR protein, leading to disease development [4,5]. This pathology affects various organs and systems, including the respiratory tract, pancreas, male reproductive system, liver, and gastrointestinal tract [6,7,8].

Cystic fibrosis is the most common hereditary disease among individuals of Caucasian descent, with a variable prevalence depending on the region. In the United States, it affects 1 in every 3500 people [9,10,11], whereas in Latin America, its incidence ranges from 1 in 1600 to 1 in 14,000 live births [12]. Historically, mortality was primarily due to respiratory complications and malnutrition caused by pancreatic insufficiency. However, therapeutic advancements have improved life expectancy, which now averages 18 years [13].

Cystic fibrosis is associated with intestinal dysbiosis, exacerbating digestive issues and contributing to chronic inflammation, which affects multiple systems and accelerates disease progression [14,15]. Dysbiosis can manifest as early as the first week of life in CF patients, and its stabilization occurs later than in healthy children, whose microbiome matures between ages 3 and 5 [16,17,18]. Additionally, CF patients exhibit lower microbial diversity, which has been linked to severe intestinal inflammation associated with *E. coli* and *P. aeruginosa*, impaired growth, increased risk of pulmonary infections, progressive decline in FEV_1_, and poorer quality of life [19,20,21].

In this regard, Frey et al. [22] reported that among 106 CF patients, those with a less diverse microbiota—dominated by pathogens such as *Pseudomonas aeruginosa*—exhibited higher levels of inflammatory markers (e.g., IL-8, IL-1β, and TNF-α) in sputum and a significantly lower FEV1, compared with patients whose microbiota was more diverse. Similarly, other studies have shown that fluctuations in the diversity of CF patients’ microbiota correlate with changes in inflammatory markers (including IL-1β and IL-8) and reductions in predicted FEV1 [23].

In this context, the use of probiotics, prebiotics, or synbiotics has emerged as a potential therapeutic strategy to restore intestinal microbiota balance and improve clinical outcomes in CF patients [24]. Prior studies suggest that probiotic supplementation may offer benefits such as decreased fecal calprotectin levels, lower risk of exacerbations, and improvements in quality-of-life indicators [25]. However, other research—for example, Weiss et al. [26]—has shown that while probiotics might reduce the rate of pulmonary exacerbations, they do not produce significant changes in pulmonary function or in inflammatory markers like IL-8 and neutrophil count. These contradictory findings could be attributed to factors such as probiotic strain, dosage, and treatment duration. Nevertheless, they underscore why, despite frequent use in CF patients [27], the effectiveness of probiotic supplementation remains debated, and why there are no solid recommendations for its routine integration into CF management.

In light of these considerations, we conducted a systematic review and meta-analysis aiming to determine the effectiveness of probiotic, prebiotic, and synbiotic supplementation in patients with CF.

## 2. Materials and Methods

### 2.1. Study Protocol

A systematic review was conducted following the guidelines of the Cochrane Collaboration Handbook [28,29] and the recommendations of the Preferred Reporting Items for Systematic Reviews and Meta-Analyses (PRISMA) [30,31]. The protocol was registered in the PROSPERO database (https://www.crd.york.ac.uk/prospero/ (accessed on 1–7 December 2024)) under the code CRD42024582172. The study was formulated according to the population, intervention, comparison, and outcomes (PICO) strategy.

### 2.2. Research Question

In patients with CF (P), does the administration of probiotics, prebiotics, or synbiotics (I), compared with placebo (C), effectively reduce pulmonary exacerbations, hospitalizations, and inflammatory markers or gastrointestinal symptoms and improve pulmonary function, quality of life, and adverse event outcomes?

### 2.3. Eligibility Criteria

#### 2.3.1. Inclusion Criteria

- Randomized Controlled Trials (RCTs).
- Trials with multiple study groups were included if the probiotic intervention arm could be isolated.
- Studies published between January 2000 and July 2024.
- Studies published in English or Spanish.
- Studies involving pediatric or adult populations with a diagnosis of CF with no restrictions regarding genotype, disease severity, or comorbidities, in which the effects of probiotics, prebiotics, or synbiotics were evaluated—regardless of treatment duration, regimen, or form of administration (capsule, food, fermented product, powder).
- Studies assessing at least one of the following outcomes: pulmonary exacerbations; hospitalizations; inflammatory biomarkers (intestinal, serological, or sputum) such as calprotectin, C-reactive protein, or cytokines; gastrointestinal symptoms; pulmonary function; quality of life; or adverse events.

#### 2.3.2. Exclusion Criteria

- Letters to the editor in which the necessary information for this review could not be extracted.
- Pre-print articles.
- Studies published solely as conference abstracts.
- Studies not available in an accessible format.
- Studies that do not specify the strain of the probiotic administered to patients.
- Investigations in which it is difficult to discern how much probiotics, prebiotics, or synbiotics contribute to the outcome compared to other simultaneous or subsequent interventions.

### 2.4. Data Sources and Search Strategy

Searches were performed in the following databases: PubMed, Cochrane Clinical Trial, SCOPUS, ScienceDirect, Web of Science, BMC, and LILAC. When necessary, filters for English and Spanish language, randomized controlled trials, and the specified time frame (March 2000 to July 2024) were applied.

The search strategy was designed and carried out independently by four researchers (SIF, JSR, SRM, DAC) from February to July 2024 using the following keywords: probiotic, prebiotic, synbiotic, Lactobacillus, Bifidobacterium, CF, and mucoviscidosis. These terms were combined with the Boolean operators AND and OR. The specific search syntax was as follows:

(“Cystic Fibrosis” OR “Mucoviscidosis” OR “Cystic Fibrosis Disease”) AND (“Probiotics” OR “Prebiotic” OR “symbiotic” OR “Lactobacillus” OR “Bifidobacterium”) AND (“Effectiveness” OR “Results” OR “Benefits”).

Adjustments were made as needed for each database. In addition, the reference lists of relevant articles were reviewed, and a manual search on the internet was performed to locate any studies not captured in the initial search. If more information was required, data were retrieved from ClinicalTrials.gov (https://clinicaltrials.gov/ (accessed on 1–7 December 2024)) or from the repository indicated in the article as the site where the clinical trial protocol was deposited. Data were managed using Rayyan—Intelligent Systematic Review (https://www.rayyan.ai/ (accessed on 1–7 December 2024)) and Zotero version 6.0 (accessed on 1–7 December 2024).

### 2.5. Selection and Extraction of Information

Two independent reviewers screened potentially eligible studies by evaluating titles and abstracts, followed by the full-text version. When access to the complete study was not possible, the corresponding author was contacted using the details provided in the publication. Studies in which eligibility remained uncertain were discussed, and inclusion decisions were reached by consensus. If an initial agreement could not be established, a third reviewer was consulted. Cohen’s Kappa index was used to measure the level of agreement among authors and to assess consistency in study selection.

Three reviewers (CLP, ANR, SIF) extracted information from the primary studies, considering the following data: main details of the clinical trial (first author, year of publication, country), participant characteristics (type of CF, number of participants per group, study population, mean age ± standard deviation, percentage of female participants), intervention characteristics (probiotic, prebiotic, or synbiotic used; product form; dose; weeks of treatment), and outcomes (pulmonary exacerbations; hospitalizations; inflammatory biomarkers in the intestine, blood, or sputum [calprotectin, C-reactive protein, cytokines]; gastrointestinal symptoms; pulmonary function; quality of life; or adverse events). Subsequently, two reviewers (YL, FCM) checked the completeness and accuracy of the recorded information.

### 2.6. Risk of Bias Assessment

Two reviewers (FCM, YLM) independently assessed the risk of bias in the included clinical trials using a standard instrument covering essential methodological aspects. The information was recorded in Review Manager version 5.4^®^ (RevMan) (accessed on 1–7 December 2024). The following criteria were evaluated:

(a) Random sequence generation
(b) Allocation concealment
(c) Blinding of participants and personnel
(d) Blinding of outcome assessment
(e) Incomplete outcome data
(f) Selective reporting

Each criterion was classified as low, unclear, or high risk of bias based on predetermined standards [29]. Discrepancies in risk-of-bias assessments were resolved through discussion until a consensus was reached.

### 2.7. Quality of Evidence Assessment

The quality of the studies included in the meta-analysis was evaluated using the Jadad scale, which assigns each study a total score ranging from 0 to 5 (indicating higher quality). The following criteria are assessed:

- Whether the study is randomized
- Whether the intervention is double-blinded
- Whether the study addresses and describes withdrawals
- Whether an appropriate randomization method is described
- Whether the inclusion and exclusion criteria are clearly described

A score of 0–2 suggests low-quality clinical trials, while a score of 3 or higher is considered the cutoff for adequate quality. No studies were excluded based on this evaluation, but their Jadad scores were taken into account in the result reporting [32].

### 2.8. Statistical Analysis

The meta-analysis was conducted using RevMan 5.4^®^ (accessed on 1–7 December 2024). Effect sizes and their respective 95% confidence intervals (CIs) were calculated. Meta-analyses were performed when three or more studies assessed an outcome relevant to this review. The analyzed outcomes included the number of patients with pulmonary exacerbations and pulmonary function. For quantitative outcomes, the mean ± standard deviation was extracted from the primary studies, applying necessary transformations if data were reported as median (interquartile range). If an outcome was measured at different time points, the final measurement in both groups was used.

Because of the nature of the outcomes, relative risk (RR) was used for dichotomous variables, and mean difference (MD) or standardized mean difference (SMD) was used for continuous variables if the same outcome was measured but reported differently (e.g., different scales or units). Outcomes not included in the meta-analysis due to variability in the data—such as hospitalizations, inflammatory biomarkers, gastrointestinal symptoms, quality of life, and adverse events—were described qualitatively. Where possible, subgroup analyses were performed based on treatment duration using six months as the cutoff. Additional subgroup analyses considered the product provided (probiotic vs. synbiotic) and the number of strains included (single vs. multiple). Subgroup analyses by age were not feasible because the articles did not differentiate results between pediatric and adult patients. A sensitivity analysis was also performed.

Statistical heterogeneity was estimated using the I^2^ statistic, with I^2^ ≥ 50% indicating high heterogeneity. Fixed-effects models were used when I^2^ was below 50%, whereas random-effects models were used when I^2^ exceeded 50%. In studies using multiple probiotic strains, a random-effects model was used regardless of the heterogeneity, considering the expected variations in outcomes. A *p*-value < 0.05 was considered statistically significant. Finally, publication bias was explored through funnel plots and Egger’s test.

## 3. Results

### 3.1. Studies Identified for the Review

**Figure 1:**
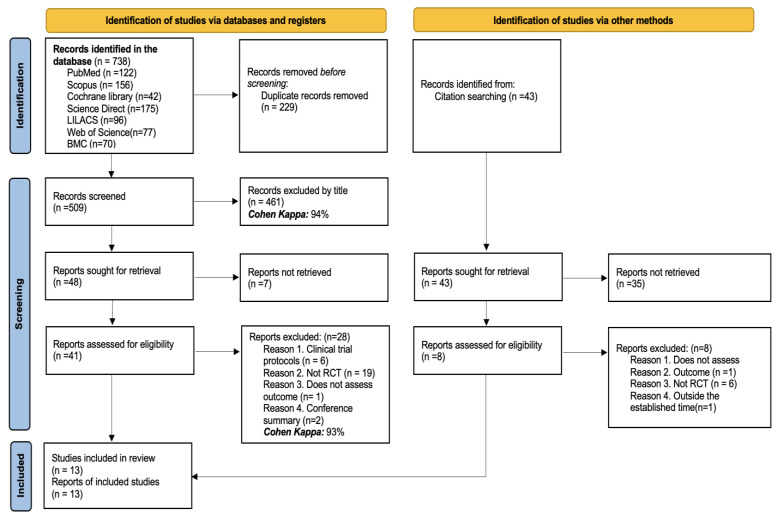
PRISMA flow diagram with the search and study selection strategy.

A total of 738 articles were identified from the database searches conducted for this review. After removing 229 duplicates, 509 articles remained. Of these, 441 were excluded based on their titles (Cohen’s Kappa: 94%). Subsequently, seven more articles were excluded after abstract review. A total of 41 full-text articles were then evaluated. Among these, 6 were excluded because they were clinical trial protocols, 19 were excluded for not being randomized controlled trials, 1 was excluded for not assessing the outcomes specified in the review, and 2 were conference abstracts. Ultimately, 13 studies were included in the systematic review (Cohen’s Kappa: 93%). A detailed outline of the study selection process is illustrated in Figure 1.

### 3.2. Characteristics of the Studies Included in the Review

Among the 13 studies included in the review, 8% were conducted before 2010, 77% between 2010 and 2020, and the remainder after 2020. In terms of geographic distribution, 38.5% took place in Iran, 30.8% in Italy, 7.7% in Brazil, 7.7% in Belgium, and 7.7% in Spain. Regarding the study populations, nine (69.2%) focused exclusively on children, whereas four (30.8%) included both pediatric and adult participants. The average sample size across studies was 40.3 participants, ranging from a minimum of 22 to a maximum of 81, with a total of 552 participants across all 13 studies. The most commonly assessed outcomes were pulmonary exacerbations (62%), pulmonary function (62%), and disease-related hospitalizations (46%). Health-related quality of life was evaluated in only two studies. See Table 1.

### 3.3. Characteristics of the Patient and Intervention

The participants assessed in the included studies showed a wide age range, with an average age of 10.7 ± 3.8 years, suggesting a predominantly pediatric population. However, some investigations also included adults, as in the case of Del Campo et al. [40], who reported an age span from 8 to 44 years.

Regarding sex distribution, most studies presented a balanced proportion of male and female participants, although not all of them provided precise sex-based statistics [44]. Only in the studies by Del campo et al. [40] and Di Nardo et al. [42] was the proportion of male participants significantly higher.

In terms of the interventions, 9 of the 13 studies (69%) used probiotics, while the remaining 4 employed synbiotics. Various Lactobacillus and Bifidobacterium strains that are known for their effects on the gut microbiota and inflammation modulation were utilized.

Among the most commonly used probiotic strains, *Lactobacillus reuteri* was widely employed, as in the studies by Tabatabai S. et al. [33] and Del Campo et al. [40]; it was administered in doses ranging from 8 × 10^8^ to 10^10^ CFU per day, either as powder or chewable tablets. *Lactobacillus rhamnosus GG* also stood out as a frequent strain; it was used in the studies by Ray K. et al. [34] and Bruzzese E. et al. [45], typically provided as 6 × 10^9^ CFU/day in capsule form. Some studies, such as that by Jafari S. et al. [43], employed combinations of several *Lactobacillus* and *Bifidobacterium* strains, including *L. casei*, *L. rhamnosus*, and *B. breve*, administered in daily capsules. Regarding synbiotics, Bilan N. et al. [36] and Freitas D. et al. used Lactobacillus/Bifidobacterium mixtures paired with fructooligosaccharides (FOSs) as the prebiotic component, given as two daily capsules in the former study and in powder form with 5.5 g/day of FOS in the latter.

Treatment duration varied considerably across studies, ranging from 1 to 12 months, which is crucial for evaluating both short- and long-term effects of the intervention. Some investigations, such as those by Bruzzese E. et al. [45] and Jafari S. et al. [43], were relatively short, lasting from 1 to 3 months. In contrast, the studies by Ray K. et al. [34] and Bruzzese E. et al. [38] implemented longer interventions of up to 12 months. See Table 2.

### 3.4. Results of the Risk of Bias Assessment

**Figure 2:**
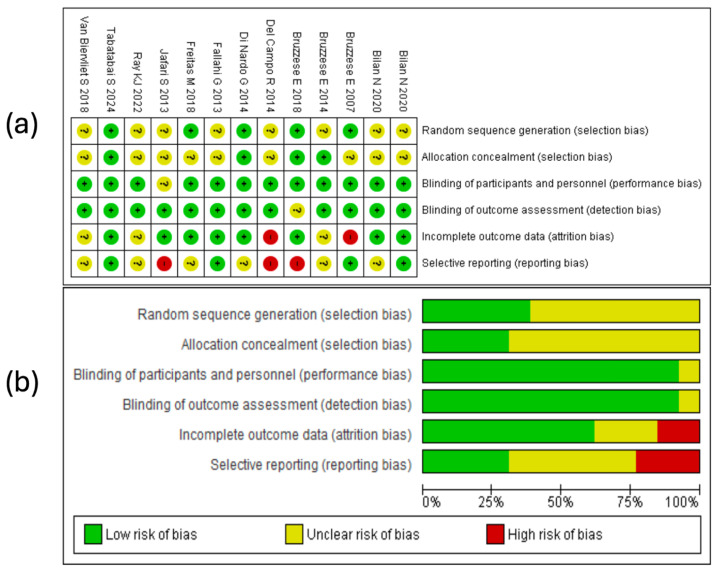
Risk of bias evaluation for the studies included in this review. (**a**) The “+” symbol denotes low risk of bias, “?” indicates unclear risk, and “–” represents high risk of bias. The colors associated with each symbol are green for low risk, yellow for unclear risk, and red for high risk. (**b**) This panel shows a summary of the identified risk of bias across all evaluated studies, displaying the percentage corresponding to each risk-of-bias item [33,34,35,36,37,38,39,40,41,42,43,44,45].

The risk of bias was analyzed by applying various criteria, as shown in Figure 2. Below is a summary of the main findings from the assessment illustrated in the graph generated in RevMan 5.4^®^ (accessed on 1–7 December 2024).

#### 3.4.1. Random Sequence Generation

Random sequence generation was classified as low risk in 5 of the 13 studies included in the systematic review [33,37,38,42,45]. Conversely, in the studies by Bilan N. et al. [35,36], Bruzzese et al. [41], Del Campo R. et al. [40], Fallahi G. et al. [44], Jafari S. et al. [43], Ray k. et al. [34], and Van Biervliet et al. [39], the risk was labeled “unclear,” as the specific procedures for randomization were not described in sufficient detail. This finding emphasizes the need for greater transparency in randomization methods to ensure proper random allocation of participants in future trials.

#### 3.4.2. Allocation Concealment

Allocation concealment (selection bias) was deemed low risk in the studies by Bruzzese E. et al. [38,41], Di Nardo G. et al. [42], and Tabatabaii et al. [33]. However, in the remaining nine studies [34,35,36,37,39,40,43,44,45], the risk was classified as “unclear” due to insufficient information regarding the measures taken to prevent investigators or participants from knowing the allocation. This finding highlights the need to document this process in detail to reduce the risk of bias.

#### 3.4.3. Blinding of Participants and Personnel

With respect to blinding of participants and personnel, most of the evaluated studies reported a low risk of bias [33,34,35,36,37,38,39,40,41,42,44,45], except for the study by Jafari S. et al. [43], in which blinding was classified as unclear. This suggests that the investigators generally implemented effective mechanisms to ensure that neither participants nor study personnel knew the intervention assignments, thereby minimizing subjective influences that could compromise the internal validity of the studies.

#### 3.4.4. Blinding of Outcome Assessment

Blinding of outcome assessment was considered low risk in most of the studies, as evidenced by Bilan N. et al. [35,36], Bruzzese et al. [41,45], Del Campo R. et al. [40], Di Nardo G. et al. [42], Fallai G. et al. [44], Freitas M. et al. [37], Jafari S. et al. [43], Ray K. et al. [34], Tabatabaii et al. [33], and Van Biervliet S. et al. [39]. Only Bruzzese E. et al. [38] was classified as unclear risk. It is important to note that when outcome assessors are not blinded, there is a possibility of less objective evaluation of clinical results in certain cases.

#### 3.4.5. Incomplete Outcome Data

Regarding incomplete outcome data, 8 of the 13 studies—such as those by Bilan N. et al. [35,36], Bruzzese et al. [38], Di Nardo G. et al. [42], Fallahi G. et al. [44], Freitas M. et al. [37], Jafari S. et al. [43], and Tabatabaii et al. [33], showed a low risk of bias, indicating no significant data losses and analyses based on complete samples. Additionally, 3 of the 13 studies showed an unclear risk [34,39,41]. In contrast, the investigations by Bruzzese et al. [45] and Del Campo R. et al. [40] exhibited a high risk of bias. Overall, these findings indicate that although most of the evaluated studies handled incomplete data adequately, certain studies presented limitations that should be considered when interpreting this review’s results.

#### 3.4.6. Selective Reporting

Regarding selective outcome reporting, most of the studies included in this review presented an unclear risk of bias, indicating uncertainty as to whether all results were properly reported [34,35,37,39,41,42]. On the other hand, 4 of the 13 studies were rated as low risk of bias [33,36,44,45]. In contrast, the studies by Bruzzese E. et al. [38], Del Campo R. et al. [40], and Jafari S. et al. [43] were classified as high risk of bias due to selective reporting. This suggests that some important findings may not have been adequately reported, potentially influencing the overall interpretation of the data.

### 3.5. Qualitative Synthesis of Results

#### 3.5.1. Hospitalizations

Out of the 13 evaluated studies, 3 examined whether probiotic products influenced hospital admissions in CF patients. In most of these studies, the administered products did not appear to reduce either the frequency or average number of hospitalizations during follow-up or even post-discharge.

In this regard, Tabatabaii et al. [33] included 40 children aged 6 to 20 years with CF, evenly distributed between an *L. reuteri* probiotic group and a placebo group. They found that in the probiotic group, one patient was hospitalized for pulmonary complications, whereas in the placebo group, there were two similar cases. No statistically significant differences were observed between the groups (*p =* 0.481). Similarly, in the study by Bruzzese et al. [38], during the first six months of follow-up, 5.3% of patients in the probiotic group versus 3% of those in the placebo group experienced at least one hospital admission (*p =* 0.641). Additionally, over the last six months of the intervention, 20.6% of those receiving *Lactobacillus rhamnosus* GG and 21.4% of those in the placebo group required at least one hospital admission, with no statistically significant difference (*p =* 0.9). Ultimately, the authors demonstrated that using this product did not significantly affect the odds of hospitalization (OR 1.67; *p =* 0.211).

In contrast, Bruzzese et al. [45] conducted a randomized controlled trial in 2007 involving 19 children who received *Lactobacillus rhamnosus GG* for six months, followed by oral rehydration solution. They observed a significant reduction in the number of hospital admissions due to pulmonary exacerbations during probiotic administration compared with oral rehydration solution (16 admissions vs. 32). Moreover, in the parallel-group analysis, children treated with the *Lactobacillus* strain showed lower hospitalization rates.

#### 3.5.2. Inflammatory Biomarkers

Seven of the thirteen studies included in this review evaluated changes in inflammatory biomarkers following probiotic or synbiotic supplementation. Of these, three examined only calprotectin; three examined calprotectin, interleukins, and rectal nitric oxide; and one focused on various cytokines such as IL-12, TNF-α, IL-10, IL-6, IL-1β, and IL-8, among others.

In their study, Ray K. et al. [34] collected stool samples from CF patients receiving a *Lactobacillus rhamnosus GG* probiotic over a 12-month period. Their findings revealed that patients whose gut microbiota was predominantly composed of *Bifidobacterium* had significantly reduced fecal calprotectin levels, suggesting lower inflammatory activity in the gastrointestinal tract. This highlights a potential relationship between gut microbiota composition and the regulation of local inflammatory processes.

Similarly, Fallahi G. et al. [44] demonstrated a significant reduction in fecal calprotectin levels after probiotic supplementation. Before the intervention, average calprotectin levels were 101 μg/g in the probiotic group and 70.22 μg/g in the placebo group. By the study’s end, calprotectin levels decreased considerably in the probiotic group compared with the placebo group (56.2 μg/g vs. 182.1 μg/g; *p =* 0.031). Additionally, Del campo R. et al. [40] reported a significant drop in fecal calprotectin levels compared with the placebo group, although no relevant differences were found in other assessed biomarkers (IL-8, IL-1β, IL-6, IL-10, TNF-α, IL-12).

Conversely, Van Biervliet et al. [39] investigated the effect of probiotics on fecal calprotectin levels in CF patients and noted that although 61% of the participants initially had elevated values (>50 mg/kg), no statistically significant changes in this inflammatory marker occurred after probiotic administration compared to the placebo group. The outcomes from other studies are summarized in Table 3.

#### 3.5.3. Quality of Life

Health-related quality of life was assessed in two investigations. Jafari S. et al. [43] examined 37 patients with CF aged 2 to 12 years, of whom 20 underwent probiotic treatment. The study showed no significant differences in total quality of life scores between the probiotic and placebo groups in reports by either children or parents. However, after three months of treatment, parents reported a significant improvement in total scores, but this difference did not persist at six months. By contrast, the children’s self-reported scores showed no significant changes at any time point. Bilan N. et al. [35], on the other hand, analyzed 40 children randomly allocated to either synbiotic supplementation or placebo for six months, measuring quality of life using the HRQOL questionnaire. Neither the total nor the sub-scores of HRQOL changed significantly following supplementation in both groups. Post-trial analyses similarly confirmed the absence of significant differences in the final scores between the synbiotic and placebo arms.

#### 3.5.4. Gastrointestinal Symptoms

Twelve of the thirteen studies in this review did not specifically evaluate the improvement of gastrointestinal symptoms after probiotic or synbiotic administration, although some noted the presence of these symptoms in the study population at baseline [33]. Del campo R. et al. [40] demonstrated a marked improvement in gastrointestinal health and inflammatory markers after probiotic use.

#### 3.5.5. Adverse Events

Three of the thirteen studies examined the safety associated with probiotic supplementation [33,40,45]. None of these trials reported serious adverse events linked to the intervention. Tabatabaii et al. [33] recorded three adverse events, of which one occurred in patients treated with *Lactobacillus reuteri*. The authors noted, however, that these events may not have been related to the product and could instead be part of the disease course.

Likewise, Bruzzese et al. [45] observed no significant side effects apart from vomiting in one child, leading to the early discontinuation of the probiotic intervention. Finally, Del Campo R. et al. [40] reported that none of the 39 patients involved in their investigation experienced adverse events.

### 3.6. Meta-Analysis Results

#### 3.6.1. Results of the Quality of Evidence Assessment

Across the analyzed studies, most met the essential criteria for randomization and adequately describing selection parameters. The studies by Tabatabai et al. [33], Bruzzese E. [38], Di Nardo G. [42], and Bruzzese E. [45] achieved the highest Jadad score of 5, indicating successful implementation of double-blinding and appropriate randomization methods. However, some studies—such as Jafari S. [43] and Ray K. [34], did not fulfill all requirements, particularly in terms of blinding and describing their randomization process. Table 4 provides more details.

A meta-analysis of two outcomes—pulmonary function and exacerbations—was performed based on the studies by Tabatabaii S. et al. [33], Di Nardo G. et al. [42], Van Biervliet et al. [39], Bruzzese E. et al. [38,41,45], Ray K. et al. [34], Freitas M. et al. [37], and Bilan N. et al. [35]. The results for each outcome are presented both globally and by subgroup, according to treatment duration, type of administered product (probiotic or synbiotic), and the number of strains used.

#### 3.6.2. Effects on Pulmonary Function

**Figure 3:**
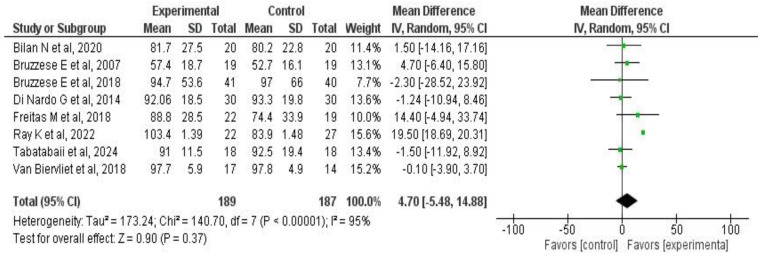
Forest plots of the effect of probiotic supplementation on FEV_1_ in patients with CF [33,34,35,37,38,39,42,45].

Eight studies [33,34,36,37,38,39,42,45] involving a total of 376 patients assessed the effects of probiotic or synbiotic supplementation on pulmonary function—primarily FEV_1_—in patients with CF. A chi-square of 140 (*p* < 0.00001) and I^2^ = 95% were observed. The meta-analysis showed that probiotic or synbiotic supplementation does not affect FEV_1_ (MD: 4.7, 95% CI: −5.4 to 14.8; *p* = 0.37) (Figure 3). A sensitivity analysis excluding the crossover trials by Biervliet et al. [39] and Bruzzese E. et al. [45] did not alter the effect of the intervention on this outcome (see Supplemental Material S1, Figures S1 and S2).

**Figure 4:**
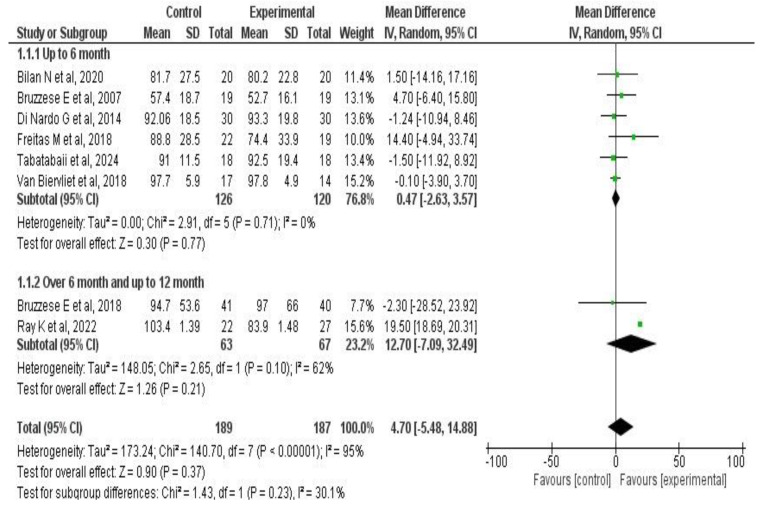
Subgroup analysis forest plots of the effect of probiotic supplementation on FEV_1_ in CF patients according to intervention duration [33,34,36,37,38,39,42,45].

A subgroup analysis was performed based on treatment duration. Among the group with treatment up to six months (*n* = 246 patients), the combined effect was MD: 0.47 (95% CI: −2.63 to 3.57; *p* = 0.77). In the subgroup treated for six to twelve months (*n* = 130), the effect was MD: 12.7 (95% CI: −7.0 to 32.4; *p* = 0.21). A comparison between these subgroups showed that the treatment effect on this outcome was consistent (*p* = 0.51). Further details are in Figure 4**.**

**Figure 5:**
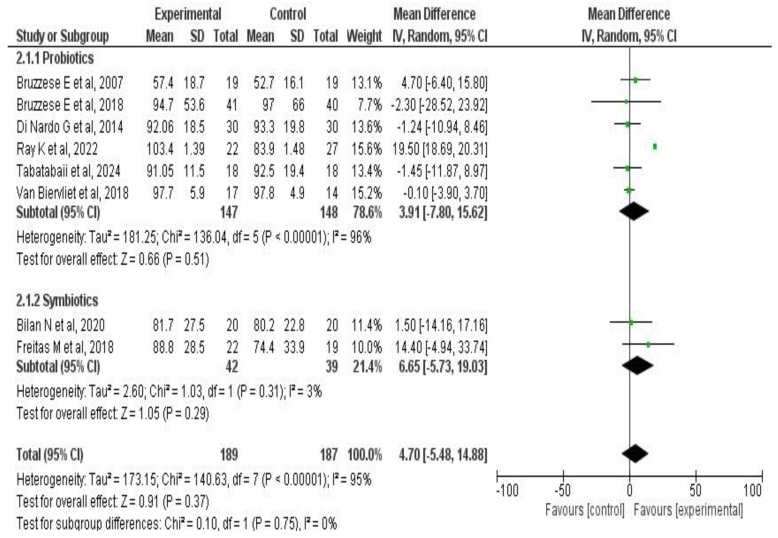
Subgroup analysis forest plots of the intervention’s effect on FEV_1_ in CF patients according to the type of administered product [33,34,36,37,38,39,42,45].

Another subgroup analysis was conducted by type of supplementation (probiotic or synbiotic). A total of 295 patients from six trials [33,34,38,39,42,45] were analyzed in the probiotic’s subgroup, yielding MD: 3.91 (95% CI: −7.8 to 15.6; *p* = 0.51). In the synbiotic subgroup, comprising 81 patients in two trials [36,37], no significant differences were found in FEV_1_ compared to standard treatment (MD: 6.6, 95% CI: −5.7 to 19.0; *p* = 0.29). Overall, the treatment effect on this outcome was similar across subgroups (*p* = 0.75). See Figure 5.

**Figure 6:**
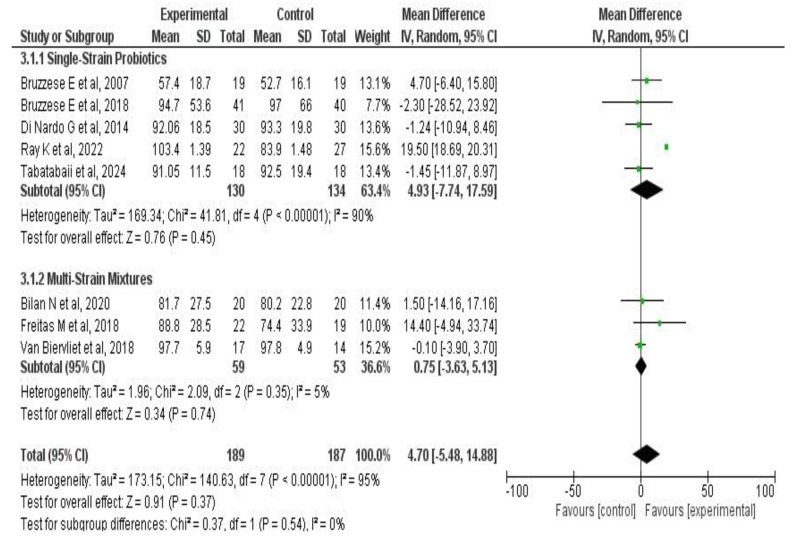
Subgroup analysis forest plots of probiotic supplementation on FEV_1_ in CF patients according to the number of used strains [33,34,36,37,38,39,42,45].

In addition, a subgroup analysis was performed based on the number of bacterial strains in the administered product. In the single-strain probiotic subgroup (five studies [33,34,38,42,45] totaling 264 patients), the effect size was MD: 4.93 (95% CI: −7.7 to 17.5; *p* = 0.45). In the multi-strain subgroup (three studies [36,37,39]), the effect was MD: 0.75 (95% CI: −3.6 to 5.1; *p* = 0.74). The results suggest that the number of administered strains does not modify the treatment effect on this outcome (*p* = 0.54). Refer to Figure 6**.**

#### 3.6.3. Effects on Pulmonary Exacerbations

**Figure 7:**
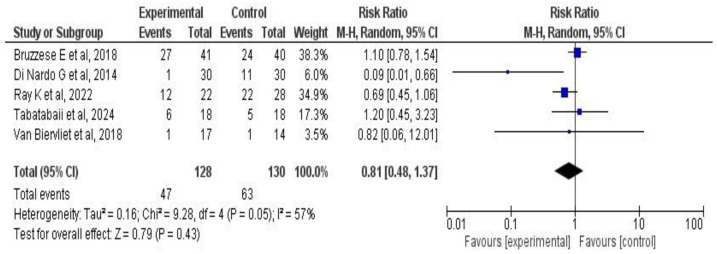
Forest plots of the effect of probiotic supplementation on pulmonary exacerbations in CF patients [33,34,38,39,42].

Five studies [33,34,38,39,42] involving a total of 258 CF patients examined the effect of probiotic or synbiotic supplementation on pulmonary exacerbations. The chi-square was 9.28 (*p* = 0.05), with I^2^ = 57%. The meta-analysis found that the intervention was not associated with changes in pulmonary exacerbations (RR: 0.81, 95% CI: 0.48–1.37; *p* = 0.43). See Figure 7.

The meta-analysis was repeated, excluding the crossover trial by Van Biervliet et al. [39], to assess potential influences of methodological design differences. Excluding this trial did not substantially change the results (RR: 0.80, 95% CI: 0.45–1.41; *p* = 0.44), suggesting a robust estimate (see Supplemental Material S1, Figure S3).

**Figure 8:**
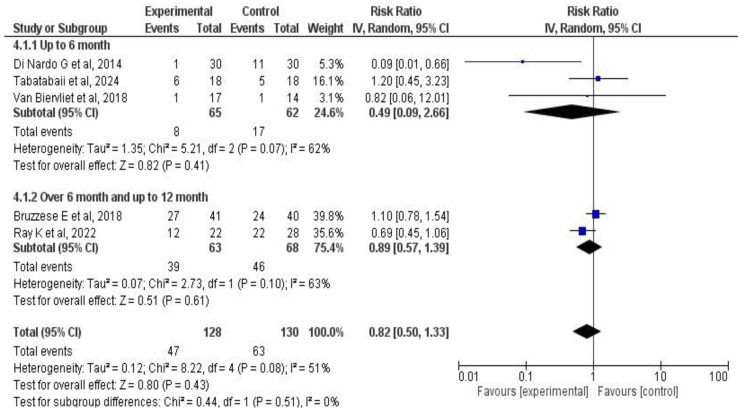
Subgroup analysis forest plots of the effect of probiotic supplementation on CF patients’ pulmonary exacerbations according to intervention duration [33,34,38,39,42].

Subgroup analysis by treatment duration showed that in the group with up to six months of treatment (*n* = 127 patients), the combined effect was RR: 0.49 (95% CI: 0.009–2.66; *p* = 0.41) with 62% heterogeneity. Conversely, in the group receiving six to twelve months of treatment (*n* = 131 patients), the effect was RR: 0.89 (95% CI: 0.57–1.39; *p* = 0.61). Overall, the treatment effect was similar across subgroups (*p* = 0.51). See Figure 8.

**Figure 9:**
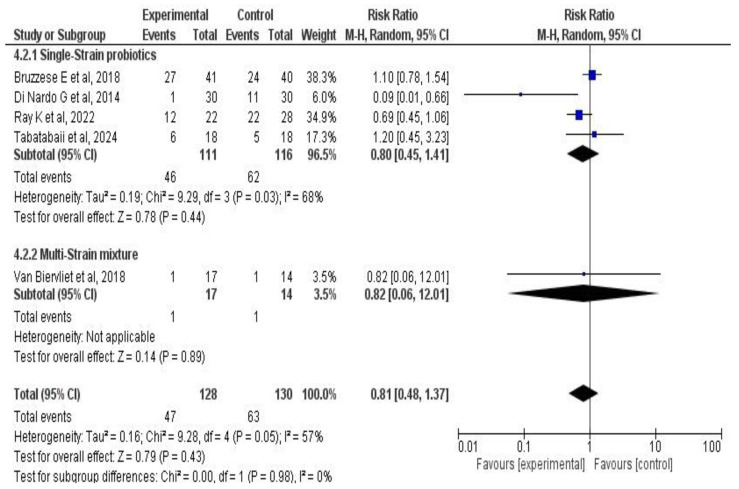
Subgroup analysis forest plots of the effect of probiotic supplementation on CF patients’ pulmonary exacerbations according to the number of used strains [33,34,38,39,42].

Additionally, another subgroup analysis was performed considering the number of bacterial strains in the administered product (single or multiple). Four studies with 227 patients made up the single-strain probiotic subgroup (RR: 0.8, 95% CI: 0.45–1.41; *p* = 0.44). The multi-strain subgroup, including the Van Biervliet et al. [39] study, showed RR: 0.82 (95% CI: 0.06–12; *p* = 0.89). The number of administered strains did not appear to modify the treatment effect on pulmonary exacerbations (*p* = 0.98). See Figure 9.

#### 3.6.4. Publication Bias

Publication bias was not assessed with a funnel plot or Egger’s test because the meta-analysis included fewer than 10 studies, which is insufficient for the reliable application of these methods. Given the limited number of studies, any observed asymmetry could be more attributable to random variation rather than genuine publication bias.

## 4. Discussion

### 4.1. Main Findings

The main objective of this review was to assess the impact of probiotic or synbiotic supplementation in patients with CF, particularly regarding pulmonary function (primarily FEV_1_), pulmonary exacerbations, quality of life, and other secondary outcomes such as hospitalizations and systemic inflammation. After analyzing 13 studies (*n* = 552) included in the systematic review, the meta-analysis found no statistically significant improvements in pulmonary function or exacerbation frequency. These findings remained consistent even after subgroup analyses based on intervention duration (up to six months vs. six to twelve months), type of product administered (probiotics vs. synbiotics), and the number of strains used (single-strain vs. multi-strain).

It is worth noting the high degree of heterogeneity (elevated I^2^) in the overall results, indicating variability among the studies in factors such as the intervention protocol, the composition of probiotic strains, the duration of treatment, and participants’ baseline characteristics. Nevertheless, within each subgroup, the direction of the effect was homogeneous, reinforcing the conclusion that, overall, supplementation does not lead to significant changes in FEV_1_ or exacerbation rates. However, some individual trials reported specific benefits in secondary outcomes, such as reductions in hospitalization rates (Bruzzese et al., 2007 [45]) or inflammatory biomarkers (Freitas et al., 2018 [37]; Del Campo et al., 2014 [40]; Fallahi et al., 2013 [44]). Yet, these observations were not robust enough to confirm a definitive global effect.

It is also important to emphasize that, on average, the 13 studies included relatively small sample sizes (40.3 participants per study) and predominantly pediatric populations, given that 9 (69.2%) of the trials focused exclusively on children. This aspect limits the extrapolation of results to adults with CF. Furthermore, most trials were conducted in a single country (e.g., Iran or Italy) or covered only local centers, which further limits the generalizability of the findings. Taken together, these factors underscore the need for larger, multicenter clinical trials with broader population representation in order to more confidently determine the potential impact of microbiota modulation through probiotics and synbiotics in cystic fibrosis.

### 4.2. Comparison with Previous Studies

The results of this study—showing no significant improvements in FEV_1_ (MD: 4.7, 95% CI: −5.4 to 14.8; *p* = 0.37) or clear reductions in pulmonary exacerbations (RR: 0.81, 95% CI: 0.48–1.37; *p* = 0.43) following probiotic or synbiotic supplementation—differ from certain previous findings. For example, Neri et al., 2019 [46] reported that four out of five trials in their review noted decreases in both the frequency of pulmonary exacerbations and intestinal inflammation, while Van Biervliet et al., 2017 [25] observed benefits in fecal calprotectin levels, the likelihood of pulmonary exacerbations, and quality of life. Nevertheless, these authors concluded—similar to the present work—that the substantial heterogeneity among studies precludes recommending probiotic supplementation as a routine intervention for CF.

Likewise, Anderson et al., 2016 [27] takes a somewhat more optimistic view regarding the potential to reduce exacerbations, citing a trend toward fewer pulmonary exacerbations with probiotic administration and reporting decreases in fecal calprotectin, a marker of gastrointestinal inflammation. However, both Anderson et al., 2016 [27] and the present study agree on the lack of a significant effect on pulmonary function (FEV_1_%).

Regarding intestinal inflammation, there is some convergence among the reviews in this manuscript; Neri et al., 2019 [46], Van Biervliet et al., 2017 [25], and Anderson et al., 2016 [27] all point to the possibility that probiotics may reduce fecal calprotectin and positively modulate the microbiota, albeit with inconsistent results. Individual studies, such as those by Del Campo et al., 2014 [40] and Bruzzese et al., 2014 [45], found significant reductions in calprotectin after using *Lactobacillus reuteri* or *Lactobacillus rhamnosus* GG, while Fallahi et al., 2013 [44] and Ray et al., 2022 [34] also identified improvements in inflammatory markers when administering synbiotics or certain *L. rhamnosus* strains. However, none of these studies demonstrated a convincing or consistent clinical benefit in pulmonary function or exacerbation rates.

Overall, the aforementioned reviews emphasize the high methodological heterogeneity across available studies, reflected in the wide variety of strains used (e.g., *L. reuteri*, *L. rhamnosus GG*, *L. paracasei*, *Bifidobacterium animalis*), the administered doses, the duration of interventions, and the methods of outcome assessment (absolute vs. percent-predicted FEV_1_; patient-reported vs. clinically recorded exacerbations). They also differ in the severity of CF and in the predominant age of the study populations, factors that could bias results. For instance, Anderson et al., 2016 [27] employed treatment durations ranging from 1 to 6 months for cohorts of up to 61 participants, whereas the present analysis included 13 studies (*n* = 552) with follow-up periods extending up to 12 months.

Despite discrepancies—particularly in relation to pulmonary exacerbations—all authors concur on the need for larger clinical trials with robust methodology and standardized protocols, aiming to definitively confirm or refute any therapeutic role of probiotics or synbiotics in CF. Extended follow-up periods are also crucial to evaluate longer-term outcomes such as overall pulmonary function, quality of life, and hospital admissions.

In a complementary vein, Al-Habsi et al., 2024 [47] found that combining probiotics with prebiotics (synbiotics) may deliver a synergistic effect on microbiota modulation and immune function. However, in the meta-analysis of this manuscript, the direct comparison between probiotics and synbiotics revealed no statistically significant differences in the clinical outcomes assessed.

### 4.3. Possible Mechanisms of Action of Probiotics and/or Synbiotics in CF

Van Dorst et al., 2022 [48] highlight the significance of the microbiome in CF and describe how probiotics can modulate its composition through mechanisms such as competition with pathogens, the production of antimicrobial substances and bacteriocins, and the enhancement of microbial stability. This perspective aligns with the review by Plaza-Díaz et al., 2018 [49], who discuss immuno-mediated mechanisms of action in pediatric intestinal diseases and note that these processes are also relevant to CF. They underscore how probiotics regulate immune responses by influencing cytokine production, activating signaling pathways like NF-κB, and interacting with Toll-like receptors, ultimately modulating both local and systemic immunity.

Additional research by Batoni et al., 2022 [50] emphasizes the role of probiotics in targeting lung pathogens in CF. These beneficial bacteria can compete with pathogens, produce antagonistic metabolites, and attenuate virulence factors while also modulating the host’s immune responses. Although Roselli and Finamore 2020 [51] address the use of synbiotics in ulcerative colitis, they report mechanisms, such as short-chain fatty acid (SCFA) production and inflammation reduction, that could be extrapolated to CF management, given the parallels in gastrointestinal and systemic immune dysregulation.

Several investigators have outlined general probiotic mechanisms relevant to CF, including interactions with the microbiota that stabilize microbial communities and suppress pathogen proliferation, immune modulation via cytokine regulation and TLR engagement, and the production of SCFAs like butyrate, acetate, and propionate. These SCFAs help maintain gut health and reduce inflammation. Probiotics also strengthen epithelial barriers and may have direct and indirect pulmonary effects through the gut–lung axis, which can potentially decrease exacerbations and enhance local mucosal defense [52,53,54,55].

Despite these theoretical benefits, Neri et al., 2019 [46] and Van Biervliet et al., 2017 [25] report that data on probiotic supplementation in CF remain limited due to small sample sizes and heterogeneous protocols. While some studies note reduced fecal calprotectin levels, improved gut microbiota composition, or lower pulmonary exacerbation frequency, Esposito et al., 2022 [24] stress that evidence is currently insufficient to recommend universal probiotic use in CF care. The strain-specific nature of probiotics, as well as variability in dosing and study duration, contributes to inconsistent clinical outcomes and underscores the need for further research to standardize interventions and define optimal therapeutic conditions.

More recent work provides additional insights into specific pathways. Price et al., 2024 [56] suggest that restoring Bacteroides populations and their propionate production can modulate inflammation and reduce the relative abundance of pathogenic bacteria in CF models, while Gur et al., 2022 [57] report potential benefits for glucose metabolism in CF-related diabetes following probiotic supplementation. Investigations by Viteri-Echeverría et al., 2024 [58] and Asensio-Grau et al., 2023 [59] show that certain probiotic strains and their synbiotic combinations positively influence colonic microbiota and relevant metabolite production. Moreover, Rodríguez-Sojo et al., 2021 [60] describe how these microorganisms can produce bacteriocins and enhance barrier integrity, and Pompilio et al., 2024 [61] demonstrate that Lactobacillus strains may inhibit *Pseudomonas aeruginosa* by reducing biofilm formation and virulence.

The connection between intestinal microbiota and pulmonary health in CF is based on the so-called “gut–lung axis,” which describes the bidirectional communication between the gut microbiota and the respiratory system [62]. Multiple studies reveal that people with CF experience gut dysbiosis early in life, characterized by reduced microbial diversity and an imbalance of species [63]. This dysbiosis typically includes a decrease in SCFA-producing bacteria, such as Faecalibacterium, Roseburia, and Akkermansia, and the proliferation of proinflammatory pathogens like *Escherichia coli*, Enterococcus, Streptococcus, Staphylococcus, Veillonella, or *Clostridium difficile*, which are favored by CF-related factors (fat malabsorption, lower intestinal pH, thick mucus, and frequent antibiotic use). These alterations can result in chronic intestinal inflammation, evidenced by increases in fecal calprotectin [64]. Numerous studies link this imbalance with worse pulmonary outcomes, notably lower FEV_1_ and a higher frequency of exacerbations [45]. Moreover, intestinal dysbiosis has been shown to predict pulmonary colonization by Pseudomonas aeruginosa, correlating with increased airway inflammation [62,63].

The mechanisms that explain how the gut–lung axis is compromised in CF include immunometabolic and anatomical pathways through which signals generated in the gut—such as cytokines and regulatory T cells—circulate systemically and modulate the pulmonary immune response. Additionally, gastroesophageal reflux and sputum swallowing may allow microorganisms or their components to migrate to the respiratory tract [65]. SCFAs produced by colonic bacteria (acetate, butyrate, and propionate) have anti-inflammatory properties and can reduce the production of proinflammatory mediators like IL-8 [66]. When this process is disrupted by dysbiosis, it fosters a proinflammatory systemic environment that worsens the pulmonary response, perpetuating a vicious cycle [63].

Therapeutic implications focus on the potential to modulate the gut microbiota as an adjunct in CF management. A more balanced diet featuring increased fiber intake and fewer saturated fats could improve microbial diversity and lessen both local and systemic inflammation [48]. Trials using probiotics have shown decreases in exacerbation frequency and inflammatory markers, though effects on pulmonary function have been variable [45]. Given the diversity of strains and the variability in treatment duration, more controlled studies are needed to draw definitive conclusions. Other approaches include prebiotics (fermentable fiber) to promote beneficial bacteria and fecal microbiota transplantation to restore the intestinal ecosystem. Additionally, CFTR modulators may indirectly influence the microbiota by enhancing mucus hydration and reducing exacerbations, opening up new opportunities for combined interventions. Overall, optimizing this gut–lung axis through the modulation of the intestinal microbiota emerges as a promising strategy to complement conventional treatments for CF [67,68].

### 4.4. Limitations of the Included Studies

The findings of this systematic review should be interpreted with caution due to the methodological and practical limitations identified in the analyzed studies. First, the small sample size in most trials—an average of 40.3 participants (range: 22–81) and with 77% of the studies enrolling fewer than 50 participants—compromises the statistical power to detect clinically relevant differences in outcomes such as pulmonary exacerbations or hospitalizations, as seen in Bruzzese et al., 2014 [41], which included only 22 patients. This limitation also prevents the exploration of benefits in specific subgroups, such as patients with homozygous F508del genotypes or severe CF.

Furthermore, the variable and sometimes insufficient duration of interventions—ranging from one month in Jafari et al., 2013 [43] to twelve months in Ray et al., 2022 [34]—makes long-term effects difficult to assess. This is crucial in a chronic disease like CF, where changes in the microbiota or pulmonary function may require years to manifest. Such heterogeneity in study designs leads to inconsistencies in the meta-analyses and limits the comparability of results.

Another concern is the risk of methodological bias: 62% of the studies did not adequately describe the methods of randomization or allocation concealment (e.g., Bilan et al., 2020 [35,36]; Van Biervliet et al., 2018 [39]), introducing selection biases. In addition, studies such as Bruzzese et al., 2018 [38] and Jafari et al., 2013 [43] omitted key data (e.g., non-serious adverse events), while Del Campo et al., 2014 [40] and Bruzzese et al., 2007 [45] reported losses to follow-up that were not addressed, raising the risk of attrition bias.

The heterogeneity of interventions is also significant: single-strain formulations (*L. rhamnosus* GG, *L. reuteri*) were used in some studies, whereas multi-strain combinations (up to eight species in Bilan et al., 2020 [35]) were used in others, with doses ranging from 10^8^ to 10^10^ CFU/day and varying formulations (capsules, powder). This diversity makes it difficult to determine the efficacy of particular strains or doses. The lack of standardized outcomes further complicates matters: only 46% of the studies evaluated hospitalizations, and only two assessed quality of life, a critical outcome in CF.

Additional limiting factors include the lack of control for confounding variables, such as concurrent antibiotic or pancreatic enzyme use—no study adjusted for these—and the absence of CFTR genotype stratification, despite the potential impact of mutations like G551D on probiotic response. Moreover, 69% of the studies were conducted in Iran and Italy, which restricts generalizability to other populations; only four studies included adults, even though CF affects all age groups.

Lastly, the underreporting of adverse events—only three studies mentioned them—and the high statistical heterogeneity in the meta-analyses (I^2^ = 95% for FEV_1_), likely stemming from variations in study designs and formulations, underscore the need for cautious interpretation. Overall, these limitations highlight the urgency for multicenter studies with larger sample sizes, longer follow-up periods, and standardized protocols to yield robust, clinically applicable conclusions.

### 4.5. Limitations of the Review

One important limitation of this review stems from the exclusive inclusion of studies published in English and Spanish, which introduces a potential language bias. Consequently, relevant research conducted in other languages may have been overlooked. The risk of publication bias is further heightened by the possibility of unpublished or non-peer-reviewed clinical trials—often referred to as gray literature—or negative results that remain absent from major databases.

In addition, the high heterogeneity observed across studies, as evidenced by elevated I^2^ values, complicated the ability to draw consistent and generalizable conclusions. Despite subgroup analyses exploring factors such as treatment duration, supplement type (probiotic vs. synbiotic), and the number of administered strains, these measures were insufficient to clarify the observed variability. This lack of clarity underscores the challenges in establishing robust recommendations.

Certain outcomes of clinical relevance—such as quality of life and inflammatory biomarkers—could not undergo a more comprehensive meta-analysis because of the small number of studies that met homogeneous criteria, as well as notable methodological diversity among them. This limitation restricts an integrated interpretation of these endpoints and highlights the necessity for future trials with consistent protocols and larger sample sizes.

Finally, the option to evaluate publication bias quantitatively through funnel plots or Egger’s test was limited by the small number of studies available for each outcome. Overall, these factors—language bias, potential unpublished data, pronounced heterogeneity, and insufficient data for certain endpoints—underline the importance of further well-designed research to strengthen the evidence base and refine clinical recommendations.

### 4.6. Clinical Implications

The approach to cystic fibrosis could be enhanced by considering the administration of probiotics and synbiotics as adjuncts to conventional treatments. Their introduction stands out as an additional option to improve the quality of care, provided that key treatment pillars—such as targeted antibiotic therapy, respiratory physiotherapy, and proper nutritional management—are upheld. If this strategy is adopted, it would be essential to precisely define the clinical indications that justify the use of these products and to establish dosing and follow-up protocols that allow for the long-term evaluation of their effects [25,27].

Another key aspect in the application of probiotics and synbiotics is the individualization of treatment. Not all patients have the same microbiota composition, nor do they exhibit the same clinical response to a specific strain. Factors like age, genetic profile, disease progression, and the presence of comorbidities can decisively influence the selection of the most suitable product. Therefore, personalizing this strategy requires a multidisciplinary approach in which pulmonologists, gastroenterologists, and nutritionists collaborate to optimize recommendations according to each patient’s particular characteristics [69,70].

Such a multidisciplinary approach also favors more comprehensive monitoring of clinical responses, allowing for adjustments if the expected benefits are not observed. In addition, collaboration among different specialties makes it possible to conduct larger-scale studies, which can help clarify the actual usefulness of probiotics and synbiotics. In the medium and long term, this perspective could provide more precise guidance regarding strain selection, supplementation duration, and how to combine these interventions with other complementary treatments [71,72].

Regarding safety, the included trials did not report any serious adverse events directly attributable to probiotics or synbiotics, which supports the feasibility of their use. However, it is crucial to maintain appropriate oversight of patient adherence, as discontinuing or inconsistently taking these products could limit their potential benefits. Consequently, educating patients and their families and offering close support are indispensable for maximizing the effectiveness of this intervention and promoting better overall control of cystic fibrosis.

### 4.7. Recommendations for Future Research

Future research in this field should include clinical trials with larger sample sizes and longer follow-up periods—ideally six to twelve months or more. This would allow for a more definitive assessment of changes in pulmonary function and exacerbation rates, as well as a clearer determination of the clinical effectiveness of probiotics or synbiotics in cystic fibrosis management. Expanding the study populations would also facilitate the application of findings to a broader range of patient ages and disease stages.

Additionally, it is advisable to integrate state-of-the-art microbiota analysis techniques, such as metagenomics and 16S rRNA sequencing. These approaches would help clarify the specific changes in gut bacterial composition and their correlation with key clinical parameters. Likewise, the uniform measurement of systemic and pulmonary inflammatory markers—including IL-6, IL-8, and calprotectin—would yield a more precise understanding of the relationship between microbiota modulation and inflammation.

Another vital consideration is the need for studies designed to compare specific strains and dosages, as well as to evaluate synbiotics versus probiotics alone. Identifying the most effective formulation would determine whether combining probiotics with prebiotics or other nutritional supplements—such as precursors of short-chain fatty acids—has a synergistic effect. Such work would contribute to optimizing therapeutic approaches in terms of both efficacy and safety.

It is essential to include additional clinically relevant endpoints to provide a more comprehensive view of the disease and the interventions. These endpoints could encompass quality of life measures, nutritional status, the Shwachman–Kulczycki score, and antibiotic-free days. Furthermore, focusing on gastrointestinal symptoms and the frequency of extrapulmonary complications would help clarify the broader impact of probiotic or synbiotic supplementation in CF.

## 5. Conclusions

This systematic review and meta-analysis found no significant improvements in FEV_1_ or reductions in pulmonary exacerbations following probiotic or synbiotic supplementation in patients with CF. Although some individual studies reported benefits in secondary outcomes, such as reduced hospitalization rates and lower inflammatory biomarkers, these findings were inconsistent and lacked sufficient robustness for clinical application. The substantial heterogeneity across studies—stemming from variations in probiotic strains, dosages, treatment durations, and population characteristics—complicates the interpretation of results and limits their generalizability, particularly to adult CF patients. Additionally, methodological limitations, including inadequate descriptions of randomization, allocation concealment, and adverse event reporting, highlight the need for more rigorous research in this area.

Despite the theoretical mechanisms supporting microbiota modulation through probiotics and synbiotics, current evidence does not justify their routine inclusion in CF management. The high variability observed in the meta-analysis underscores the necessity for well-designed, multicenter, randomized controlled trials with standardized protocols to ensure methodological consistency. Future research should explore strain-specific efficacy, alternative administration routes such as aerosolized probiotics, and stratification based on CFTR genotype and disease severity to identify potential subgroups that may benefit from these interventions. Until more conclusive evidence is available, probiotics and synbiotics should be considered experimental adjuncts rather than established therapeutic options in CF treatment.

## Figures and Tables

**Table 1:** Characteristics of the studies included in the review.

Author, Year	Country	Disease	Population Included	Number of Patients	Outcomes Evaluated
Tabatabai S. et al., 2024 [33]	Iran	CF	Children and adults	40	Pulmonary exacerbations, hospitalizations, pulmonary function, and composition of the gastrointestinal microbiota.
Ray KJ. et al., 2022 [34]	Italy	CF	Children	49	Pulmonary exacerbations, hospitalizations, pulmonary function, inflammatory biomarkers, and intestinal microbiota composition.
Bilan N. et al., 2020 [35]	Iran	CF	Children	40	Health-related quality of life.
Bilan N. et al., 2020 [36]	Iran	CF	Children	36	Pulmonary exacerbations, hospitalizations, and pulmonary function.
Freitas D. et al., 2018 [37]	Brazil	CF	Children	41	Pulmonary function and inflammatory biomarkers.
Bruzzese E. et al., 2018 [38]	Italy	CF	Children	81	Pulmonary exacerbations, hospitalizations, and pulmonary function.
Van Biervliet. et al., 2018 [39]	Belgium	CF	Children	31	Pulmonary function, pulmonary exacerbations, and inflammatory biomarkers.
Del campo R et al., 2014 [40]	Spain	CF	Children and adults	30	Inflammatory biomarkers, intestinal microbiota composition, quality of life, and adverse effects.
Bruzzese E. et al., 2014 [41]	Italy	CF	Children	22	Inflammatory biomarkers and intestinal microbiota composition.
Di Nardo G. et al., 2014 [42]	Italy	CF	Children and adults	60	Pulmonary exacerbations, hospitalizations, inflammatory biomarkers, and pulmonary function.
Jafari S. et al., 2013 [43]	Iran	CF	Children	37	Pulmonary exacerbations and health-related quality of life.
Fallahi G. et al., 2013 [44]	Iran	CF	Children	47	Inflammatory biomarkers.
Bruzzese E. et al., 2007 [45]	Italy	CF	Children and adults	38	Pulmonary exacerbations, hospitalizations, and pulmonary function.

CF: cystic fibrosis.

**Table 2:** Essential characteristics of the patients included in each trial and the therapy administered.

Author, Year	Patients (I/C)	Male (%)	Age Range (Years)	Mean Age	Product (Type, Formulation, Dose, Administration Route)	Placebo	Treatment Duration (Months)	Conclusion
Tabatabai S. et al., 2024 [33]	*n* = 36 I: 18 C: 18	42.5	6–20	12	Probiotic. *Lactobacillus reuteri* (8 × 10^8^ CFU). One sachet per day, orally.	NE	4	No significant changes were observed in forced expiratory volume in one second (FEV_1_), forced expiratory flow at 25–75%, or forced vital capacity between the two groups after the treatment period.
Ray K.J. et al., 2022 [34]	*n* = 49 I: 22 C: 27	52	2–16	7.4 *	Probiotic. *Lactobacillus rhamnosus GG.* Daily supplementation orally.	NE	12	Patients receiving probiotic supplementation achieved better clinical outcomes compared to the control group.
Bilan N. et al., 2020 [35]	*n* = 36 I: 18 C: 18	55	5–12	8.75	Synbiotic. *L. casei* (3.5 × 10^9^ CFU/g), *L. acidophilus* (1 × 10^9^ CFU/g), *L. rhamnosus* (7.5 × 10^8^ CFU/g), *L. bulgaricus* (10^8^ CFU/g), *Streptococcus thermophilus* (1 × 10^8^ CFU/g), *B. breve* (1 × 10^10^ CFU/g), *B. longum* (3.5 × 10^9^ CFU/g), and 38.5 mg of fructooligosaccharides. Two capsules daily, orally.	Maltodextrin	6	The probiotic blend did not reduce pulmonary exacerbations, did not significantly improve pulmonary function, and did not reduce the number or duration of hospitalizations.
Bilan N. et al., 2020 [36]	*n* = 40 I: 20 C: 20	52	5–12	8.72	Synbiotic. *L. casei* (3.5 × 10^9^ CFU/g), *L. acidophilus* (1 × 10^9^ CFU/g), *L. rhamnosus* (7.5 × 10^8^ CFU/g), *L. bulgaricus* (10^8^ CFU/g), *S. thermophilus* (1 × 10^8^ CFU/g), *B. breve* (1 × 10^10^ CFU/g), *B. longum* (3.5 × 10^9^ CFU/g), and 38.5 mg of fructooligosaccharides. Two capsules daily, orally.	Maltodextrin	6	After six months of synbiotic supplementation, there was no significant effect on health-related quality of life in CF patients.
Freitas et al., 2018 [37]	*n* = 41 I: 22 C: 19	53.6	1–16	10.1	Synbiotic. Fructooligosaccharides 5.5 g/day; *L. paracasei*, *L. rhamnosus*, *L. acidophilus*, and *B. lactis* (10^8^–10^9^ CFU/day each strain). Powder, orally.	Maltodextrin	3	Synbiotic supplementation showed promise in reducing the pro-inflammatory markers IL-6 and IL-8. No differences were found between groups in FEV_1_ levels or nutritional status.
Bruzzese E. et al., 2018 [38]	*n* = 81 I: 41 C: 40	50.6	2–16	8.5	Probiotic. *Lactobacillus rhamnosus GG* (6 × 10^9^ CFU/day). Capsule, orally.	Maltodextrin	12	*Lactobacillus rhamnosus GG* supplementation did not significantly improve respiratory or nutritional outcomes in the studied CF population.
Van Biervliet et al., 2018 [39]	*n* = 31 I: 17 C: 14	42	6.9–12	9.3	Probiotic. *Lactobacillus rhamnosus* SP1 (DSM 21690) and *Bifidobacterium animalis* spp. BLC1 (LGM23512), 10^10^ CFU/day, orally.	NE	4	Probiotic supplementation did not influence fecal calprotectin, pulmonary function, pulmonary exacerbations, or the microbiome.
Del campo R. et al, 2014 [40]	*n* = 30 I: 15 C: 15	70	8–44	17.7 *	Probiotic. *Lactobacillus reuteri* DSM17938 (10^8^ CFU). Chewable tablet, 1 tablet/day, orally.	NE	6	Probiotics may improve CF-related gut microbiota dysbiosis characterized by a high density of Proteobacteria. *L. reuteri* reduces intestinal inflammation and improves digestive comfort.
Bruzzese E. et al., 2014 [41]	*n* = 22 I: 10 C: 12	59	2–9	7 *	Probiotic. *Lactobacillus rhamnosus GG* (6 × 10^9^ CFU/day). Capsule, orally.	Maltodextrin	1	*L. rhamnosus GG* administration can partially restore a healthy gut microbiota, favoring communities that limit intestinal inflammation and improving the disease course.
Di Nardo G. et al., 2014 [42]	*n* = 60 I: 30 C: 30	65	6–29	17.5	Probiotic. *Lactobacillus reuteri* ATCC55730 (10^10^ CFU). Five drops/day, orally.	NE	6	*L. reuteri* reduces pulmonary exacerbations and upper respiratory tract infections in CF patients with mild to moderate pulmonary disease. Its administration may exert a beneficial effect on the course of CF.
Jafari S. et al., 2013 [43]	*n* = 37 I: 20 C: 10	35	2–12	5.3	Probiotics. *L. casei*, *L. rhamnosus*, *S. thermophilus*, *B. breve*, *B. infantis*, and *L. bulgaricus* (each at 10^9^ CFU). Two capsules per day, orally.	NE	1	Probiotics may help reduce the number of pulmonary exacerbations and improve quality of life in CF patients, though these quality-of-life improvements appear to be temporary.
Fallahi G. et al., 2013 [44]	*n* = 47 I: 24 C: 23	NE	NE	8.56	Synbiotic. Fructooligosaccharide plus a bacterial mix at 1 × 10^9^ CFU per sachet, including *L. casei*, *L. rhamnosus*, *S. thermophilus*, *B. breve*, *L. acidophilus*, *B. infantis*, and *L. bulgaricus.* One sachet daily, orally.	Maltodextrin	1	About two-thirds of the patients had intestinal inflammation. Probiotic administration was shown to reduce calprotectin levels and thereby decrease intestinal inflammation in CF patients.
Bruzzese E. et al., 2007 [45]	*n* = 38 I: 19 C:19	42	5–23	13.2	Probiotic. *Lactobacillus rhamnosus GG* (6 × 10^9^ CFU/day). Solution, orally.	ORS	6	*L. rhamnosus GG* reduced pulmonary exacerbations and hospital admissions in CF patients, suggesting that probiotics may delay respiratory failure and that a connection exists between intestinal and pulmonary inflammation.

* Median; NE: not specified; CFU: Colony-Forming Units; ORS: oral rehydration solution; CF: cystic fibrosis.

**Table 3:** Behavior of the reported inflammatory biomarkers.

Author, Year	Patients (I/C)	Product (Formulation and Dose)	Evaluated Biomarkers	Sample Type	Results on Inflammatory Biomarkers
Ray K.J. et al., 2022 [34]	*n* = 49 I: 22 C: 27	Probiotic. *Lactobacillus rhamnosus* GG. Daily supplementation, oral route.	Calprotectin	Fecal	The use of *Lactobacillus rhamnosus* GG was associated with a higher frequency of Bifidobacterium-dominant microbiota, which led to lower fecal calprotectin levels.
Freitas et al., 2018 [37]	*n* = 41 I: 22 C: 19	Synbiotic; 5.5 g/day fructooligosaccharides (FOSs); *L. paracasei*, *L. rhamnosus*, *L. acidophilus*, *B. lactis* (10^8^–10^9^ CFU/day per strain). Powder, oral route.	IL-12, TNF-α, IL-10, IL-6, IL-1β, IL-8, MPO, NOx	Serum	Significant differences in NOx levels were found after supplementation with the product. In the group that received synbiotics with positive bacteriological results, reductions in IL-6 (*p* = 0.033) and IL-8 (*p* = 0.009) were observed.
Van Biervliet et al., 2018 [39]	*n* = 31 I: 17 C: 14	Probiotic. *Lactobacillus rhamnosus* SP1 (DSM 21690) and *Bifidobacterium animalis* ssp. BLC1 (LGM23512), 10^10^ CFU/day, oral route.	Calprotectin	Fecal	Although 61% of the patients initially showed abnormal calprotectin values, there were no statistically significant changes in this marker following probiotic supplementation.
Del campo R. et al., 2014 [40]	*n* = 30 I: 15 C: 15	Probiotic. *Lactobacillus reuteri* DSM17938 (10^8^ CFU). Chewable tablet, 1 tablet/day, oral route.	Calprotectin, IL-8, IL-1β, IL-6, IL-10, TNF-α, IL-12	Fecal	CF patients who received probiotic supplementation showed lower calprotectin levels compared to the placebo group (20.3 vs. 33.8 μg/mL; *p* = 0.003). No differences were noted in other biomarkers.
Bruzzee E. et al., 2014 [41]	*n* = 22 I: 10 C: 12	Probiotic. *Lactobacillus rhamnosus GG* (6 × 10^9^ CFU/day). Capsule, oral route.	Calprotectin, rNO	Fecal	Children with CF had elevated fecal calprotectin and rectal nitric oxide levels compared to healthy controls. *Lactobacillus GG* administration reduced fecal calprotectin levels and partially restored the gut microbiota in CF patients.
Di Nardo G. et al., 2014 [42]	*n* = 60 I: 30 C: 30	Probiotic. *Lactobacillus reuteri* ATCC55730 (10^10^ CFU). Five drops/day, oral route.	Calprotectin, TNF-α, IL-8	Fecal, Serum	No statistically significant differences were found between the groups in fecal calprotectin or the measured cytokines (TNF-α, IL-8).
Fallahi G. et al., 2013 [44]	*n* = 47 I: 24 C: 23	Synbiotic. Fructooligosaccharides plus a bacterial mix at 1 × 10^9^ CFU per sachet, containing *L. casei*, *L. rhamnosus*, *S. thermophilus*, *B. breve*, *L. acidophilus*, *B. infantis*, *L. bulgaricus.* One sachet/day, oral route.	Calprotectin	Fecal	At baseline, patients in the probiotic group had an average fecal calprotectin level of 101 μg/g, whereas those in the placebo group had 70.22 μg/g. After probiotic supplementation, levels in the intervention arm were significantly lower than in the placebo group (56.2 μg/g vs. 182.1 μg/g; *p* = 0.031).

IL-12: interleukin-12; TNF-α: tumor necrosis factor-alpha; IL-10: interleukin-10; IL-6: interleukin-6; IL-1β: interleukin-1β; IL-8: interleukin-8; MPO: myeloperoxidase; NOx: nitric oxide metabolites; rNO: rectal nitric oxide; CF: cystic fibrosis.

**Table 4:** Quality of evidence assessment using the Jadad scale.

Author	The Study Is Randomized	The Intervention Is Double-Blind	Study Withdrawals Are Accounted for and Described	The Randomization Procedure Is Adequate	Selection Criteria	Score
Tabatabai S. 2024 [33]	1	1	1	1	1	5
Ray K. 2022 [34]	1	1	0	0	1	3
Freitas M. 2018 [37]	1	1	0	1	1	4
Bruzzese E. 2018 [38]	1	1	1	1	1	5
Jafari S. 2013 [43]	1	0	1	0	1	3
Del Campo R. 2014 [40]	1	1	1	0	1	4
Bruzzese E. 2014 [41]	1	1	0	0	1	3
Fallahi G. 2013 [44]	1	1	1	0	1	4
Di Nardo G. 2014 [42]	1	1	1	1	1	5
Bruzzese E. 2007 [45]	1	1	1	1	1	5
Bilan N. 2020 [36]	1	1	1	0	1	4
Bilan N. 2020 [35]	1	1	1	0	1	4
Van Biervliet S. 2018 [39]	1	1	1	0	1	4

## Data Availability

The raw data supporting the conclusions of this article will be made available by the authors on request.

## Supplementary Materials

The following supporting information can be downloaded at: https://www.mdpi.com/article/10.3390/medicina61030489/s1, Figure S1: Sensitivity analysis of the effect of probiotic supplementation on FEV_1_ in patients with cystic fibrosis, excluding the Van Biervliet study; Figure S2: Sensitivity analysis of the effect of probiotic supplementation on FEV_1_ in patients with cystic fibrosis, excluding the Bruzzese E study; Figure S3: Sensitivity analysis of the forest plots on the effect of probiotic supplementation on pulmonary exacerbations in CF patients, excluding the Van Biervliet study. PRISMA checklist.

## Abbreviations

CF	Cystic fibrosis
*CFTR*	*Cystic Fibrosis Transmembrane Conductance Regulator*
CFU	Colony-Forming Units
