# Supplementary material for: Effectiveness of Probiotics, Prebiotics, and Symbiotic Supplementation in Cystic Fibrosis Patients: A Systematic Review and Meta-Analysis of Clinical Trials
Source: Medicina (Kaunas). 2025 Mar 12;61(3):489. doi: 10.3390/medicina61030489 (PMC11944062; doi:10.3390/medicina61030489)
Supplement: Supplementary file 1 [file medicina-61-00489-s001.zip › Supplemental Materials.pdf]

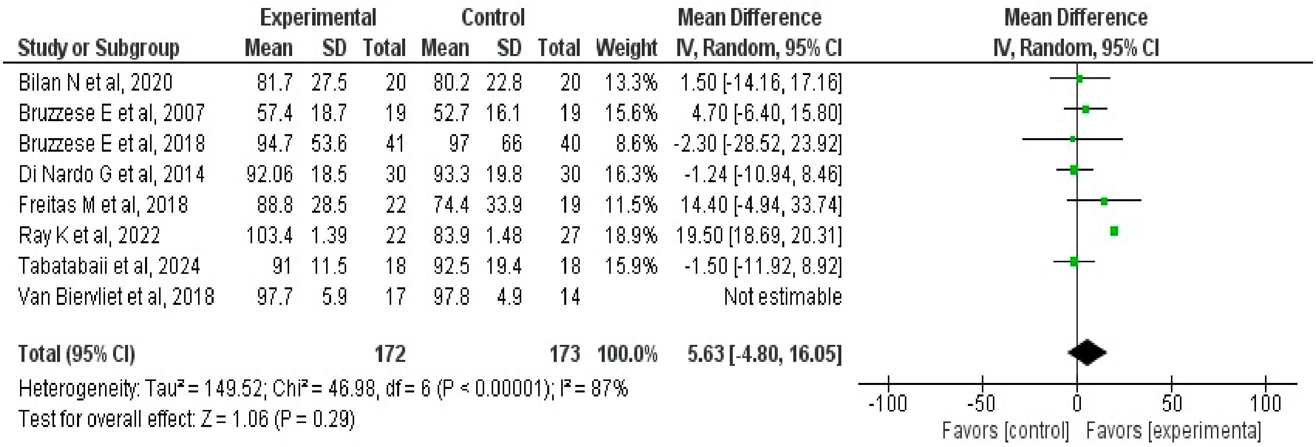


**Supplemental Material Figure S1. Sensitivity analysis of the effect of probiotic supplementation on $FEV_1$ in patients with cystic fibrosis, excluding the Van Biervliet study.**

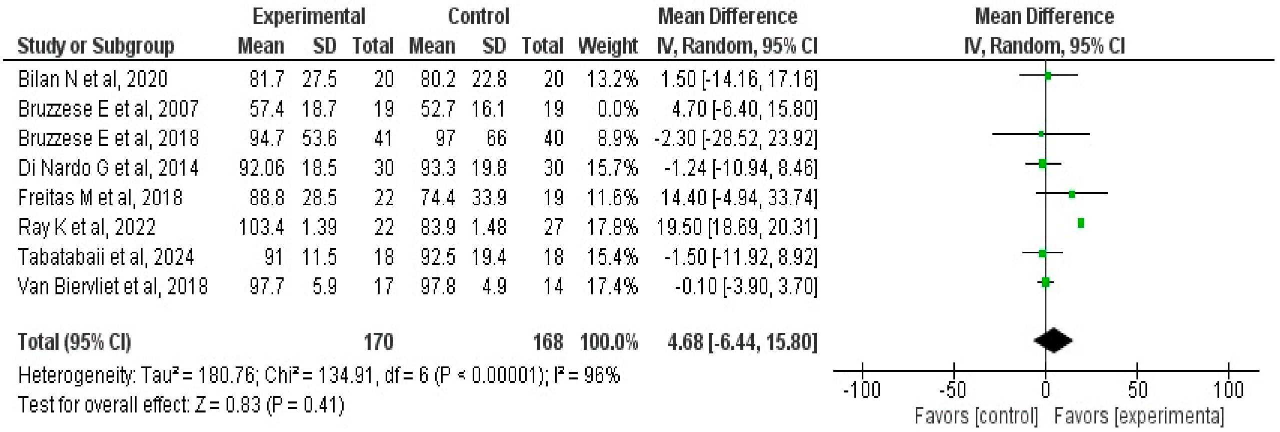


**Supplemental Material Figure S2. Sensitivity analysis of the effect of probiotic supplementation on $FEV_1$ in patients with cystic fibrosis, excluding the Bruzzese E study.**

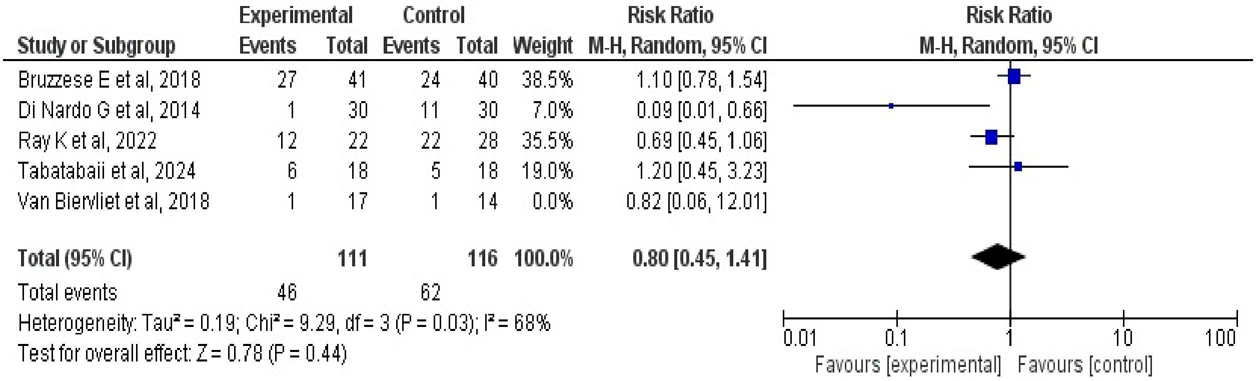


**Supplemental Material Figure S3. Sensitivity analysis of the forest plots on the effect of probiotic supplementation on pulmonary exacerbations in CF patients, excluding the Van Biervliet study.**
